# Notch1 hallmarks fibrillary depositions in sporadic Alzheimer’s disease

**DOI:** 10.1186/s40478-016-0327-2

**Published:** 2016-07-01

**Authors:** Emanuele Brai, Noemi Alina Raio, Lavinia Alberi

**Affiliations:** Unit of Anatomy, Department of Medicine, University of Fribourg, Route de Gockel, 1, Fribourg, 1700 Switzerland; Unit of Pathology, Department of Medicine, University of Fribourg, Route de Gockel, 1, Fribourg, 1700 Switzerland; Swiss Integrative Center for Human Health, Passage du Cardinal, 13B, Fribourg, 1700 Switzerland

**Keywords:** Notch1, Sporadic Alzheimer’s disease, Amyloid plaques, Tau, Cerebrospinal fluid

## Abstract

**Background:**

Notch1 signaling is a cellular cascade with a fundamental role from brain development to adult brain function. Reduction in Notch1 affects synaptic plasticity, memory and olfaction. On the other hand, Notch1 overactivation after brain injury is detrimental for neuronal survival. Some familial Alzheimer’s disease (FAD) mutations in Presenilins can affect Notch1 processing/activation. Others report that Notch1 is overexpressed in sporadic Alzheimer’s disease (AD). These works indicate that imbalances in Notch1 may be implicated in AD pathophysiology. In this study, we addressed whether Notch1 alteration can be considered a hallmark of AD.

**Results:**

Immunohistochemical analysis of Notch1 on cortical and hippocampal tissue from post-mortem patients indicates an accumulation of Notch1 in plaque-like structures in the brain parenchyma of subjects with sporadic AD. Further analysis shows that displaced Notch1 is associated with fibrillary tangles/plaques. Biochemical validation confirms an accumulation of Notch1 in cytosolic brain fractions. This increase in protein is not accompanied with a raise in the Notch1 targets Hes1 and Hey1. Examination of the cerebrospinal fluid (CSF) indicates that the full length and truncations of the Notch1 protein are reduced in AD patients hinting at an accumulation in the brain parenchyma.

**Conclusions:**

Our research indicates that Notch1 is significantly displaced and accumulated in fibrillary structures in the susceptible hippocampal and cortical regions of sporadic AD patients. The dominant deposition of Notch1 in the brain parenchyma and its general signal reduction in neurons is consistent in all the AD patients analyzed and suggests that Notch1 may potentially be considered a novel hallmark of AD.

**Electronic supplementary material:**

The online version of this article (doi:10.1186/s40478-016-0327-2) contains supplementary material, which is available to authorized users.

## Introduction

Alzheimer’s disease is the most common form of dementia. The latest Alzheimer’s Disease International (ADI) data, presented during the World Alzheimer Report 2015, indicate that there are approximately 46 millions people affected by dementia worldwide. This number is expected to increase with the rising lifespan to 131.5 millions by 2050.

Alzheimer’s disease is mainly characterized by specific hallmarks such as amyloid plaques, formed by insoluble A *β*42 aggregates, and neurofibrillary tangles (NFTs), due to Tau hyperphosphorylation inducing microtuble disassemble. Progression of AD is staged according to the spread of hyperphosphorylated Tau mapping the cerebral progression of the disease. Stages are grouped in three general units: from healthy (I-II) to mild cognitive impairment (III-IV) and finally to definite AD (V-VI) [1, 2]. According to the Braak staging of AD, hyperphosphorylated Tau spreads from the transentorhinal cortex (stages I-II), to the limbic allocortex and adjacent neocortex (stages III-IV) and finally to the neocortex affecting primary and secondary areas [3]. There are two forms of AD, the early-onset AD, also known as familial AD (FAD), and the late-onset AD or sporadic AD. The early-onset AD is caused by missense genetic mutations in amyloid precursor protein (APP), presenilin (PS) 1 and 2, all impinging on APP processing and leading to an increase in A *β*42 generation [4]. FAD accounts for 5 % of the total AD cases. In contrast, sporadic AD represents the predominant form of the disease. The major risk factor for sporadic AD is the genetic variant Apolipoprotein E4 (ApoE4) [5]. Furthermore, genome wide association studies have identified single nucleotide polimorphisms (SNPs) in genes adjacent to the ApoE locus, which encode for proteins involved in lipid metabolism and inflammation [6–8].

Despite the great efforts in understanding the causes of AD, it remains largely unresolved which are the pathophysiological triggers involved in the development of neurodegeneration. Most studies have relied on genetic FAD models involving APP and Presenilins’ mutations [9] rather than addressing the sporadic form of the disease. Only most recently APP knockin mouse models [10] as well as neuroinflammation model, by administration of Polyriboinosinic:polyribocytidylic acid (PolyI:C) [11] and lipopolysaccharides (LPS) [12], have been used to investigate long-term changes recapitulating the progression of the sporadic form. In our study, we took advantage of the mouse model of neuroinflammation (PolyI:C) [11]. When administered prenatally, PolyI:C, a synthetic analog of viral dsRNA, evokes a chronic neuroinflammatory state in the offspring accompanied by amyloidogenic APP processing in the aged hippocampus and a stark memory deficit [11]. Based on the late-onset of the AD-like symptoms, this mouse model appeared the most suitable to investigate changes in Notch1 signaling, particularly, taken that this intracellular cascade has been previously implicated in dementia [13]. The Notch proteins are highly conserved transmembrane receptors, with pleiotropic functions from neuronal development to organ homeostasis. The Notch receptors are activated via ligand binding [14]. The ligand-receptor association dimerizes the receptor and triggers sequential cleavages by metalloproteases (ADAM10/ADAM17) [15] and the gamma secretase complex [16]. These proteolytic events generate a Notch intracellular domain with nuclear signaling potential. Notch receptors, besides activating transcription (canonical pathway) [17], can also operate in a non-transcriptional way (non canonical pathway) [18] and determine their function in a context-specific manner [19].

Among the Notch receptors, the best studied is the Notch1 homologue. In the last two decades, several studies, using transgenic flies, mice and also rats, have demonstrated that Notch1 displays a fundamental role in mature brain function [20]. In the adult mammalian brain, Notch1 is highly expressed in neuronal progenitors as well as pyramidal neurons of the cortex and hippocampus, which are the most sensitive to neurodegeneration. Lower levels are also reported in glia cells [21].

In adult neural stem cells, Notch1 contributes to neurogenesis and neuronal maturation. On the other hand, in postmitotic neurons, Notch1 is involved in spinogenesis [22] and synaptic efficacy [22, 23]. At network level, Notch1 can be induced and activated by an increase in synaptic activity [22] and influences plasticity regulating information processing.

Loss of function murine models of Notch1 share a memory formation deficit [22, 24] and a more recent study indicates that Notch1 is also essential for olfaction [25]. Interestingly, olfaction and memory are brain functions, that are progressively affected in dementia [26, 27] making the case for a possible involvement of Notch1 in the neurological deficits associated with the disease. Further studies have indicated that, following ischemic or epileptic injury, an aberrant increase in Notch1 expression contributes to neuronal demise [28–30] and neuroinflammation [31]. Moreover, Notch1 imbalances have been reported in patients affected by Alzheimer’s disease [32, 33], fronto-temporal dementia and Down’s syndrome [34]. Despite there is no common consensus on whether Notch1 signaling is augmented or reduced in dementia, all these studies suggest a potential involvement of this signaling pathway in the progression of the diseases. In particular, studies on FAD have shown that mutations in Presenilin 1 (PS1) and Presenilin 2 (PS2) can reduce Notch signaling [33, 35]. In contrast, another report indicates that, in sporadic AD, Notch1 expression is increased [32]. Moreover, a clinical study using Samagacestat, an inhibitor of gamma secretase, has recently failed due to worsening of cognitive function, to be attributed most probably to Notch1 dysfunction [36].

Based on our own studies indicating a critical balance between physiological or pathological Notch1 functions [20], we performed a careful comparative study of Notch1 expression, activation and signaling on post-mortem specimen from sporadic AD patients and age-matched controls. Our study shows that Notch1 expression is fundamentally disrupted in AD patients with accumulation in fibrillary plaques and tangles. On the other hand, both Notch1 expression and activation are sensibly reduced in neurons. Moreover, analysis of Notch1 in CSF indicates that extent of clearance of Notch1 is significantly decreased in AD patients. This is the first study, which reports a significantly altered pattern of Notch1 in human AD brains with potential for a pathophysiological role of Notch1 in the progression of AD.

## Materials and methods

### Human tissue

The human samples were generously provided from the Brain Bank for Dementia Research, Oxford, UK. We received paraffin embedded brain sections and frozen brain tissue from the entorhinal cortex of 5 controls and 5 sporadic AD patients. Furthermore, from a second group of 6 non demented controls and AD patients, we obtained paraffin embedded liver sections and frozen cerebrospinal fluid (CSF). The use of human tissue has been approved by the Ethical commission of the Brain Bank for Dementia UK (OBB ID N. TW344 and OBB ID N. TW296) and the Swiss Ethical Commission of the Canton Vaud and Fribourg (N.32514) for the use of human samples. All experiments conducted on human tissue comply to the WMA Declaration of Helsinki.

### Animals

Floating sagittal sections from control (NaCl) and immunochallenged (PolyI:C) mice [11] were obtained from Dr. Knuesel (Institute of Pharmacology, University of Zuerich). Sagittal sections were from 22 months old NaCl (*n* =2) and PolyI:C (*n* =2) mice.

### Cells

Human breast carcinoma cells, MDA-MB-231, expressing high levels of Notch1 (gift of Dr. Del Sal, University of Trieste) were cultured in DMEM (PAA, Austria) supplemented with 10 % fetal bovine serum (PAA, Austria), glutamine and penicillin/streptomycin (Invitrogen, USA). 12 hours before harvesting the media, the cells were changed to a DMEM-based serum free media.

### Antibodies and labeling reagents

The primary antibodies used for the chromogen immunohistochemistry on brain sections were polyclonal goat anti-Notch1, which recognizes the C-terminal of the protein, 1:500 (sc-6014; Santa Cruz Biotechnology, USA) and rabbit anti-cleaved Notch1 (NICD), 1:200 (cat. no. 2421; Cell Signaling, USA). The secondary antibodies and the other reagents are the same as previously described [25]. The primary antibodies for the immunofluorescence were polyclonal goat anti-Notch1 against the C-terminus, 1:500 (sc-6014; Santa Cruz Biotechnology, USA), goat anti-Notch1 extracellular portion, 1:500 (sc-23299; Santa Cruz Biotechnology, USA), rabbit anti-Notch1 cytoplasmic domain, 1:500 (cat. no. 07-220; Millipore, USA), rabbit anti-APP, which recognizes the C-terminal of the protein, 1:500 (ab2073; Abcam, UK), mouse anti-CD68, 1:150 (NBP2-29406; Novus, UK), rabbit anti-CD68, 1:500 (sc-9139; Santa Cruz Biotechnology, USA), rabbit anti-GFAP, 1:5000 (IS52430, Dako, USA), rabbit anti-phosphorylated Tau, 1:500 (phospho T205/ab4841; Abcam, UK), rabbit anti-A *β*42, 1:250 (cat. no. 8243; Cell Signaling, USA), mouse anti-A *β*42, 1:2000 (cat. no. 05-831-I; Millipore, USA), mouse anti-NF200, 1:500 (cat. no. 1178709; Boehringer Mannheim Biochimica, Germany). We also used Thioflavin T (ab120751; Abcam, UK) to stain the misfolded *β* sheets at a concentration of 100 mM diluted in water, following the manufacturer’s instructions. Immunofluorescence has been also performed on liver sections using goat anti-Notch1 against the C-terminus, 1:500 (sc-6014; Santa Cruz Biotechnology, USA), rabbit anti-A *β*42, 1:250 (cat. no. 8243; Cell Signaling, USA), goat anti-Notch1 extracellular portion, 1:500 (sc-23299; Santa Cruz Biotechnology, USA), and rabbit anti-Notch1 cytoplasmic domain, 1:500 (cat. no. 07-220; Millipore, USA). The primary antibodies utilized for the western blot analysis on entorhinal cortex sections and CSF were goat anti-Notch1, 1:500 (sc-6014; Santa Cruz Biotechnology, USA), goat anti-Notch1 extracellular portion, 1:500 (sc-23299; Santa Cruz Biotechnology, USA), rabbit anti-Notch1 cytoplasmic domain, 1:500 (cat. no. 07-220; Millipore, USA), rabbit anti-phosphorylated Tau, 1:1000 (phospho T205/ab4841; Abcam, UK), mouse anti-PSD95, 1:1000 (sc-32290; Santa Cruz Biotechnology, USA), rabbit anti-NeuN, 1:1000 (ab177487; Abcam, UK), mouse anti- *β* actin, 1:2000 (sc-81178; Santa Cruz Biotechnology, USA), mouse anti-Gapdh, 1:8000 (sc-365062; Santa Cruz Biotechnology, USA). The secondary antibodies used for the immunofluorescence were Cy3 donkey anti-goat (cat. no. 705-165-147), Cy5 donkey anti-mouse (cat. no. 715-605-150), Cy2 donkey anti-rabbit (cat. no. 711-545-152). All fluorescent conjugated antibodies were purchased from Jackson Immunoresearch Europe Ltd and were all diluted 1:1000. The secondary antibodies used for the immunoblots were infrared-dye-conjugated (IR-Dye) from LI-COR Biosciences GmbH, Germany and were donkey anti-mouse IgG IR800 (cat. no. 926-32212;), donkey anti-mouse IgG IR680 (cat. no. 926-68072), donkey anti-rabbit IgG IR800 (cat. no. 926-32213) and donkey anti-goat IgG IR 800 (cat. no. 926-32214). All IR-antibodies were diluted 1:10 000.

### Immunohistochemistry

Chromogen immunohistochemistry, to detect the expression and distribution of Notch1 and its cleaved fragment (NICD1), was done on healthy and AD patients sections. Prior to starting the immunolabelings, human sections were deparaffinized in xylol [3 × 10 minutes (min)] and rehydrated in decreasing concentrations of ethanol [2 × 100 %, 2 × 96 %, 1 × 80 %, 1 × 70 % and 2 × distilled water for 5 min each]. After this step, human and mice sections were treated following the same protocol. Antigen retrieval was performed warming the sections with 10mM of sodium citrate buffer (pH 6), for 45 min at 65 °C in a water bath. Thereafter, sections were washed 3 × 5 min with Trizma-based solution (TBS), then 1 × 10 min with TBS containing 0.1 % Triton and then blocked for 1 hour at room temperature (RT) with a blocking solution (TBS containing 10 % fetal bovine serum (FBS) and 0.1 % Triton). Primary antibodies were diluted in TBS with 1 % FBS and 0.1 % Triton, distributed dropwise to cover the section and let incubating overnight at 4 °C. Floating murine sections were incubated on a shaker with gentle agitation, whereas human specimens were kept inside a wet histochamber standing inside the refrigerator. The next day, sections were washed three times for 5 min and then incubated for 3 hours at RT with secondary antibodies diluted in the same buffer used for primary antibodies. After this incubation, 3 × 5 min washes with TBS preceeded, in some staining, the use of Thioflavin T, which was followed by 2 × 5 min washes with distilled water. Then the sections were incubated for 10 min with DAPI and washed for 2 × 5 min with TBS. In the next step, the human sections, were treated with Sudan Black B for 5 min to eliminate the autofluorescence caused by lipofuscin accumulation in aged cells and then dipped in 70 % clean ethanol until reaching the desired level of staining. After, the sections were washed 2 × 5 min with TBS and mounted with an aqueous mounting media.

### Imaging quantification

Brain sections were imaged using a slide scanner (NanoZoomer, Hamamatsu, Japan) with 40 × objective and a confocal microscope (Leica TCS SP5; Leica Microsystems GmbH, Germany) with 40 × and 63 × objectives. The intensity of the Notch1 and NICD1 signals was performed on chromogen 3,3’-Diaminobenzidine (DAB) immunolabeling from randomly selected ROIs on the dorsal hippocampus (CA1-CA2 region) and entorhinal cortex. The Notch1 and NICD1 signals were extracted from the mask of each neuron using TrakEM2 (Image J, NIH, Bathesda, USA) and the signal was processed using Matlab (Additional file 1: Figure S1A-C’). For Notch1, pixel quantification was performed by setting the constraint for the brown color (brown = 50 >= red <= 255 and 20 >= green <= 100 and 0 >= blue <= 90) within the tracked soma. The intensity of the Notch1 signal was analyzed on hippocampal (205 cells for AD and 242 for the control group) and cortical (239 cells for AD and 165 for the control group) neurons. For the NICD1, ROIs were selected as for Notch1 and quantification was performed by using the Euclidean distance weight function of the NICD1 pixels from a reference parameter within the soma of hippocampal (187 cells for AD and 180 for the control group) and cortical (141 cells for AD and 148 for the control group) neurons. The euclidean distance is therefore inversely proportional to the signal strength.

The plaques countings were performed using the sections imaged with the Nanozoomer at 40 × magnification. A 4 × 6 square grid, covering an area of 12.1 *μm*^2^ was chosen as region of interest (ROI) and was randomly superimposed on the images at a constant magnification. The countings were conducted manually and blindly for each patient and immunofluorescence staining. For the dorsal hippocampus as well as the entorhinal cortex (Additional file 1: Figure S1D), 15 ROIs were chosen. Plaques positive for Notch1, A *β*42, p-Tau, Thioflavin T and double immunolabeled were counted. Plaques with a diameter larger than 30 *μ*m were considered only (Additional file 1: Figure S1E-E”). In the liver, Notch1 intracellular and Notch1 extracellular immunoreactivity was measured post-hoc as mean grey value in ROIs, drawn on the perimeter of the hepatocytes. An average of 10 ROIs in 5 randomly selected areas of the liver were selected and analyzed per patient.

### Immunoblotting

Western blot analysis was performed on whole cell lysates, synaptosomal, cytosolic, nuclear fractions of the entorhinal cortices, and cerebrospinal fluids from either healthy controls or Alzheimer’s patients’.

#### Whole cell lysates

To obtain whole cell lysate, the tissue was fragmented on dry ice and homogenized with non-ionic NP-40 buffer, containing proteases’ inhibitors cocktail, 1:100 (3749.1, Roth, Germany) with the addition of Pepstatin A 5mM (2936.2, Roth, Germany) and phosphatase inhibitors, such as Sodium Orthovanadate,1:100 (450243, Sigma-Aldrich, USA), *β*-glycerophosphate disodium salt hydrate, 1:100 (G9422, Sigma-Aldrich, USA), Sodium Fluoride, 1:50 (201154, Sigma-Aldrich, USA). Briefly, the tissue was homogenized using plastic pestels and thereafter sonicated (Bandelin Sonopuls HD 70, Berlin), with 10/1 second pulses. Thereafter, the samples were centrifuged at 1000 g for 10 min at 4 °C. Then, the supernatant was transferred to a new tube. The protein concentration was measured through the BCA procedure (Roth, Germany) and then samples were stored at −80 °C until use.

#### Subcellular fractionation

The entorhinal cortex was homogenized with plastic pestles by adding Hepes/Sucrose solution, proteases and phosphatases inhibitors (PPI) and sonicated as above indicated. After homogenization, in each tube was added Hepes/Sucrose to have a final Vol. of 1.4-1.5 ml. Then, samples were centrifuged at 1000 g for 10 min at 4 °C to remove the nuclear fraction and unbroken cells (pellet, N) from the other fractions (supernatant, S1). The pellet was further resuspended in high-salt Hepes Buffer (5 mM HEPES, 1.5 mM MgCl2, 0.2 mM EDTA, 0.5 mM DTT, 300 mM NaCl and 26 % glycerol (v/v), pH 7.9). After further homogenization with a pestel, the sample was left on ice for 30 min and then centrifuged at 21,000 g for 25 min at 4 °C. The resulting supernatant containing the nuclear lysate was aliquoted and stored at –80 °C. The supernatant (S1) from the first spin was transferred in a new tube and centrifuged at 10,000 g for 15 min. The supernatant (S2) was collected. The pellet (P1) was resuspended in 10 Vol of Hepes/Sucrose buffer and centrifuge at 10,000 g for 15 min. Thereafter, the resulting pellet (P2) (crude synaptosomal fraction) was lysed by hyposmotic shock in H2O (200 *μ*l ddH2O + PPI) and rapidly adjusted to 4 mM Hepes (200 *μ*l Hepes + PPI) and mixed constantly for 30 min on a rotating wheel at 4 °C. The protein concentration for the cytoplasmic component S2 and the synaptosomal fraction P2 was evaluated through the BCA procedure (Roth, Germany) and samples were stored until use at −80 °C.

#### Cerebrospinal fluid preparation

Cerebrospinal fluid samples were thawed on ice, resuspended in a commercial loading buffer (PCG3009, Sigma-Aldrich, USA) and then heated either at 95 °C for 10 min or 50 °C for 15 min [37]. 25 *μ*l of each sample were loaded per lane.

#### Western blot analysis

Brain tissue lysates or CSF were denatured using *β*-mercapto-ethanol-based loading buffer. Proteins were separated using standard electrophoresis and western blot procedure. Depending on the molecular weight of the protein of interest, 8–10 % custom-made gels or precast gels 4–12 % (PCG2015, Sigma-Aldrich, USA) were used. For the precast PAGE 4–12 % gels, a commercial running buffer was used (PCG3001, Sigma Aldrich USA). After running, the proteins were transferred to a nitrocellulose membrane (cat # 04530301, Membrane Solutions, Germany) using a semidry transfer machine (Trans Blot Turbo, BioRad) for the tissue samples, or a wet transfer for the CSF. The membranes were incubated for 1 hour with a blocking solution containing 2 % bovine serum albumin (BSA) (cat # 3737.2, Roth, Germany) in TBST buffer. Afterwards, the membranes were probed, overnight, with primary antibodies at 4 °C, with gentle agitation. The next day, the membranes were washed 5 × 5 min with TBST, then incubated for 1 hour, with infrared dye-conjugated secondary antibodies (LiCOR, Germany) diluted in TBST. The membranes were then rinsed 6 × 5 min with TBST and let to air dry, covered by aluminum foil. Finally, the infrared labeled proteins were revealed using an Infrared scanner (LiCOR, Germany).

### Statistical analysis

Quantification of Notch1 immunoreactivity and NICD1 expression in the soma of hippocampal and cortical neurons was performed using unpaired Student’s T-test. From the fluorescent immunolabeled sections, the average number of plaques positive for Notch1, A *β*42, Thioflavin T, or p-Tau over 15 randomly selected areas was analysed for normality within each group. Gaussian normal distribution of the data was confirmed using the Shapiro-Wilk test and the Student’s T-test was subsequently applied. The *α* significance level was considered 0.05 with a *p*-value said to be significant when being <0.05. The consistency of the counting for Notch1 in all three stainings was confirmed using the Kruskal-Wallis test. From the result, a post-hoc power analysis was performed. The number of neurons showing signs of cell cycle re-entry was counted on 10 captions obtained from randomly selected areas in hippocampus and entorhinal cortex from each patient. Differences between healthy controls or AD were analyzed with one-way ANOVA. For the Western Blot analysis, the optical density of each band was assessed through Image J software and normalized with Gapdh for the cytoplasmic component (S2), with PSD95 for the synaptic fraction (P2), with NeuN for the nuclear partition (N) and to the healthy control bands (CSF). A paired Student’s T-test was performed to assess differences between healthy controls and AD samples.

## Results

### Notch1 is deposited in amyloid plaques

**Fig. 1:**
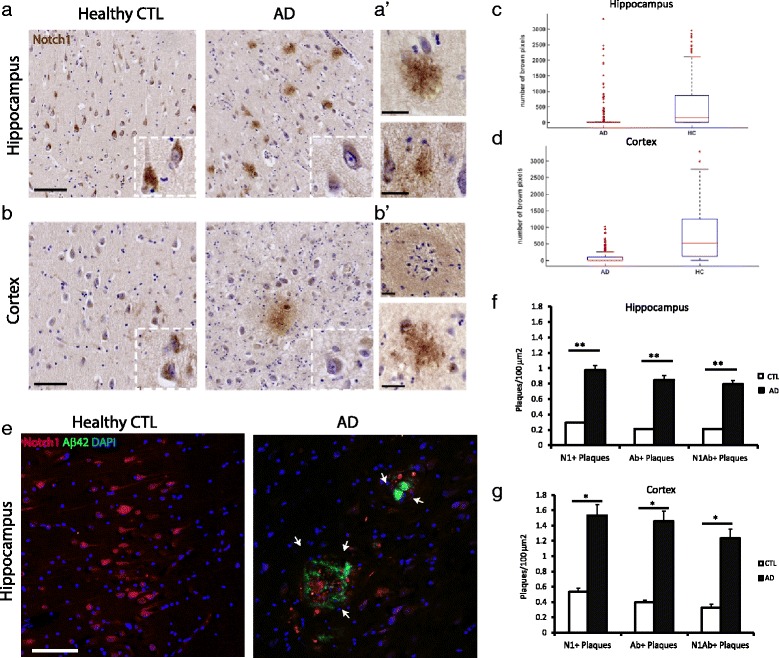
Characterization of Notch1 expression in sporadic AD brain tissue. **a**–**b**’) Immunohistochemical analysis on hippocampus (**a**–**a**’) and entorhinal cortex (**b**–**b**’) shows an aberrant Notch1 deposition in the brain parenchyma. Moreover, in AD patients, Notch1 labeling appears fainter in the cell bodies and processes of cortical and hippocampal neurons as compared to healthy controls (**a**–**b**). **a**’-**b**’) Notch1 positive plaques with different conformation either core plaques (upper captions) or diffuse distribution (bottom captions). **c**–**d**) Box plots summarizing the quantification of Notch1 positive pixels occupying the soma of (**c**) hippocampal and (**d**) cortical neurons of AD and healthy control patients (*p*<0.001 for both regions). **e**) Double immunofluorescence analysis on the dorsal hippocampus indicates that, in AD tissue, Notch1 is deposited in A *β*42 positive plaques and is decreased in neuronal soma as compared to healthy controls. Nuclei have been counterstained using DAPI. **f**) Bar graph showing the counting of plaques/100 *μ* *m* ^2^, in the hippocampus, positive for Notch1, A *β*42 and double positive. **g**) Bar graph showing the counting of plaques per 100 *μ* *m* ^2^, in the entorhinal cortex, positive for Notch1, A *β*42 and double positive. *= *p*<0.05, **= *p*<0.01. Error bars are SEM. Scale bars are 100 *μ*m in (**a**) and (**e**), and 25 *μ*m in (**a**’) and (**b**’)

Previous studies have reported alteration in Notch1 expression in human AD patients [32, 33]. However, there is no agreement on whether Notch1 is reduced or increased. In this study we investigated the pattern of Notch1 expression in post-mortem brain, CSF and liver tissue from sporadic AD patients and age-matched healthy controls. The average age of the AD patients and healthy controls (CTL) was 80 ± 9.9 and 79.6 ± 9.2, respectively. Patients were of both genders. The AD patients were staged according to the Braak Scale to an average of 5.4 and the healthy CTLs to 1.4 (Table 1). Using paraffin embedded sections comprising the entorhinal cortex and the hippocampus, we performed chromogen immunolabeling using a Notch1 antibody, which recognizes the C-terminal tail (Fig. 1a and b). On the healthy controls sections, we observed a homogeneous labeling of Notch1 in the soma and processes of both hippocampal and cortical pyramidal neurons. On the other hand, in the AD patients, we found that Notch1 was delocalized to the parenchyma in plaque-like structures (Fig. 1a and b). Moreover, Notch1 expression in neurons of the CA1 hippocampal region (Fig. 1a/inserts and Fig. 1c) and cortex (Fig. 1b/inserts and Fig. 1d) was significantly reduced (*p*<0.001). Notch1 was present in different plaques’ formation from core to diffused aggregates (Fig. 1a’ and b’). Plaques positive for Notch1 could also be found in the healthy controls, but at a much lower frequency (Fig. 1f and g). The Notch1 pattern resembled our earlier findings using the PolyI:C mouse model of AD [11], in which we found visible aggregates of Notch1 in the brain parenchyma of the hippocampus and cortex (Additional file 2: Figure S2A-A”). Similarly to the human AD samples, the expression of Notch1 in neurons from the PolyI:C mice was generally reduced as compared to the age-matched NaCl injected controls (Additional file 2: Figure S2A”). In order to characterize the Notch1 deposition and to address whether Notch1 accumulates in amyloid plaques, we performed double fluorescent immunohistochemistry (FIHC) using Notch1 and A *β*42 antibodies. We observed that Notch1 can be found in amyloid rosette structures mostly in a complementary pattern with A *β*42 and only scattered overlapping puncta (Fig. 1e). Confirming the chromogen staining, the FIHC shows that there is a general reduction of Notch1 immunoreactivity in pyramidal neurons of the CA1 hippocampal region and a displacement in abnormal aggregates (Fig. 1e). Analysis of the plaques in the dorsal hippocampus (CA1-CA2 region) and entorhinal cortex (Fig. 1f and g) reveals that the majority of such aggregates is positive for both Notch1 and A *β*42. However, a minor portion appears positive only for Notch1 (Additional file 3: Figure S3, white arrows) or only A *β*42 (Fig. 3a’, light blue arrows) (Fig. 1f and g). Correlation analysis indicates that the magnitude of A *β*42 and Notch1 positive depositions is comparable in the examined areas (*R*=0.98, Hippocampus; *R*=0.95, Entorhinal Cortex).

**Fig. 2:**
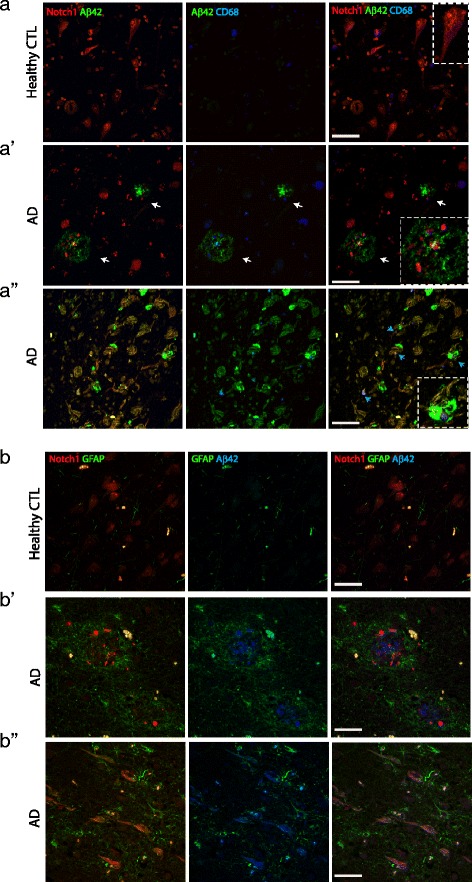
Notch1 is expressed in A *β*42 positive plaques invaded by activated microglia and astroglia. **a**–**a”**) Fluorescent immunolabeling reveals that in AD patients, A *β*42 and Notch1 are present in core plaques (arrows in **a’** and magnified insert) and fibrillary-like plaques (**a”**) with abundant activated microglia, CD68 positive. In (**a’**) a magnified insert shows a microglia (blue) ensheathed in an amyloid fibrillary Notch1 positive plaque. In the healthy controls (**a**), A *β*42 and CD68 immunoreactivity is significantly lower and Notch1 labels homogeneously the neuronal soma (insert). **b**–**b”**) Representative fluorescent immunolabeling shows that in AD patients, core plaques and fibrillary structures positive for Notch1 and A *β*42 are invaded by GFAP positive astroglia. GFAP immunoreactivity is significantly lower in healthy controls (**b**) as compared to the AD samples **b’**-**b”**). Scale bars in all panels are 40 *μ*m

**Fig. 3:**
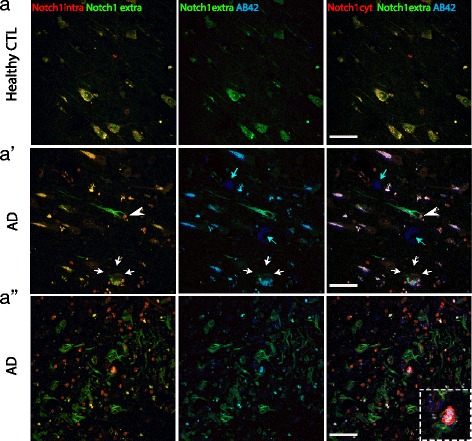
Notch1 intracellular and extracellular domains have distinct patterns in AD brains. **a**–**a**”) Fluorescent immunolabeling indicates that the Notch1 extracellular domain is abundantly expressed in the processes and is co-distributed with the Notch1 cytoplasmic fragment on the cell body of healthy neurons (**a**). In degenerating neurons (arrowhead in **a**’), the extracellular domain of Notch1 is strongly expressed in the soma and process, whereas the cytoplasmic portion of the receptor is condensed into the nucleus (arrowhead in **a**’). In addition, the two domains of Notch1 co-label some A *β*42 stained plaques (white arrows in **a**’ and insert in **a**") but not all (light blue arrows in **a**’). Notably, the Notch1 extracellular portion is strongly present in the amyloid fibrils, while the intracellular component is present at a lower level (**a**"). Scale bars in all panels are 40 *μ*m

**Table 1:** Human specimen

Patient ID	Age	Sex	Braak staging	CERAD	Tissue	Applications	
035/13	85	m	5	Definite AD	Entorhinal Cortex	IHC/WB/qPCR	
016/12	64	f	6	Definite AD	Entorhinal Cortex	IHC/WB/qPCR	
089/11	83	f	6	Definite AD	Entorhinal Cortex	IHC/WB/qPCR	
133/12	78	m	6	Definite AD	Entorhinal Cortex	IHC/WB/qPCR	
100/12	90	m	4	Probable AD	Entorhinal Cortex	IHC/WB/qPCR	
1117/00	91	f	6	Definite AD	Liver/CSF	IHC/WB	
C4295	84	m	6	Definite AD	Liver/CSF	IHC/WB	
142/05	87	m	6	Definite AD	Liver/CSF	IHC/W	
C4298	76	m	5-6	Definite AD	Liver/CSF	IHC/WB	
C4130	79	f	5-6	Definite AD	Liver/CSF	IHC/WB	
036/02	69	f	6	Definite AD	Liver/CSF	IHC/WB	
169/11	84	f	2	Normal	Entorhinal Cortex	IHC/WB/qPCR	
071/13	83	f	2	Normal	Entorhinal Cortex	IHC/WB/qPCR	
136/12	77	f	1	Normal	Entorhinal Cortex	IHC/WB/qPCR	
121/12	89	f	2	Normal	Entorhinal Cortex	IHC/WB/qPCR	
048/12	65	f	1	Normal	Entorhinal Cortex	IHC/WB/qPCR	
048/02	89	m	1	Normal	Liver/CSF	IHC/WB	
1085/03	67	m	1	Normal	Liver/CSF	IHC/WB	
053/06	71	m	1	Normal	Liver/CSF	IHC/WB	
083/01	79	f	0	Normal	Liver/CSF	IHC/WB	
1050/00	81	f	1	Normal	Liver/CSF	IHC/WB	
1182/94	80	f	1	Normal	Liver/CSF	IHC/WB	

*CERAD*, Consortium to Establish a Registry for Alzheimer’s Disease

To validate the neuroinflammatory potential of Notch1 and amyloid plaques, we performed the triple immunolabeling with the activated microglia marker, CD68 (Fig. 2a–a”). We observed that double labeled core aggregates were always invaded by CD68-positive microglia (Fig. 2a’, insert). Interestingly, some microglia were also positive for Notch1. The presence of Notch1 in microglia was confirmed in the PolyI:C model (Additional file 2: Figure S2B, white arrowhead), suggesting that an imbalance of Notch1 may increase the pro-inflammatory potential of those cells [31, 38]. In the healthy control sections, activated microglia were present but to a lower extent (Fig. 2a). CD68-positive microglia were also infiltrating fibrillary-like aggregates positive for Notch1 and A *β*42 (Fig. 2a”, insert). As expected, the immunoreactivity for A *β*42 in the entorhinal cortex and hippocampal CA region of healthy controls appeared very low (Fig. 2a and b). To further address whether Notch1 deposits trigger an astroglia reaction, we performed triple FIHC with Notch1, A *β*42 and the glial fibrillary acidic protein (GFAP). We observed that, in the AD samples, core plaques positive for Notch1 and A *β*42 (Fig. 2b’) were surrounded by GFAP-positive astroglia. Interestingly, fibrillary-like Notch1 positive aggregates were ensheated in astroglia processes (Fig. 2b”). Several astroglia were also positive for Notch1, both in healthy and AD sections (Fig. 2b–b”), indicating that Notch1 function is not exclusive to neurons and may also play a role in astrogliosis. As expected the presence of astroglia cells in the healthy controls section was very low (Fig. 2b).

In order to validate the presence of Notch1 in amyloid aggregates, we carried out the labeling of Notch1 using two antibodies directed towards the extracellular portion of Notch1 (Notch1 extra) and the intracellular PEST domain of Notch1 (Notch1 intra) (Fig. 3a–). We observed that the two antibodies had overlapping patterns, labeling nicely CA1 pyramidal neurons in the healthy controls (Fig. 3a). On the other hand, in the AD samples the extracellular Notch1 intensively labeled the processes of a neuron, which presents a condensed nucleus with staining only for the Notch1 intracellular domain (Fig. 3a’, white arrowhead). Moreover, the Notch1 extracellular domain antibody shows strong overlap with A *β*42 implying that the N-terminus domain of Notch1 may be released and deposited in some (Fig. 3a’, white arrows) but not all amyloid aggregates (Fig. 3a’, light blue arrows). Fibrillary-like depositions were intensively stained for Notch1 extracellular and were labeled with Notch1 intracellular (Fig. 3a”), suggesting the presence of the native full protein in these aggregates. A *β*42 labeling was more scattered on those process-like structures and overlapped with Notch1 extracellular and Notch1 intracellular (Fig. 3a”, insert). Notch1 intracellular fragment labeled several nuclei (Additional file 4: Figure S4A), likely corresponding either to glia or degenerated neurons.

**Fig. 4:**
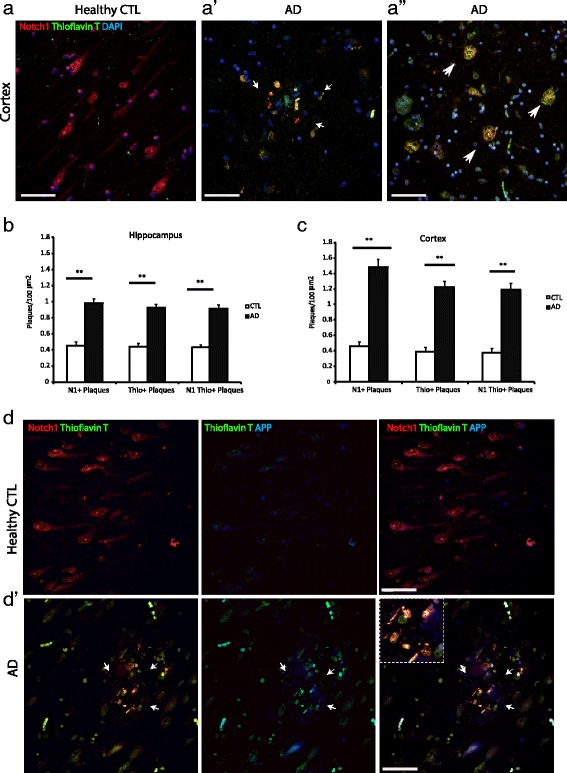
Notch1 distribution in Thioflavin T positive plaques and fibrillary aggregates. **a**–**a”**) Double immunofluorescence indicates the co-expression of Notch1 and Thioflavin T stained fibrils in AD brains (**a’**-**a"**) as compared to the age matched control (**a**). In non-demented patients, Notch1 is homogeneously distributed in neurons and Thioflavin T signal is not present. **b** and **c**) Bar graphs showing the counting, of single or double labeled plaques for Notch1 and Thioflavin T in the hippocampus (**b**) and in the entorhinal cortex (**c**). **d**-**d’**) Fluorescence immunostaining of the entorhinal cortex for Notch1, APP and Thioflavin T shows that Notch1 is mainly localized with Thioflavin T deposits (arrows in **d’** and insert) and to a lower extent with APP in AD brains. Expression of Notch1 and APP is critically altered in AD brains as compared to healthy control tissue (**d**). **= *p*<0.01. Error bars are SEM. Scale bars are 50 *μ*m in all images

To confirm the fibrillary nature of the Notch1 aggregates, we performed double counterlabelings of Notch1 immunostained sections with Thioflavin T and DAPI (Fig. 4 and Additional file 4: Figure S4B). In AD patients, we observed an overlap between Notch1 and Thioflavin T in the radius of plaques, but not in their core (Fig. 4a’, white arrows), as well as some but not all fibrillary-like aggregates (Fig. 4a”, large white arrows). Thioflavin T and Notch1 depositions were observed also in healthy controls but at lower frequency in both areas (Fig. 4b and c). Correlation analysis confirms the coincidence of Notch1 and Thioflavin T in plaque depositions (*R*=0.95, dorsal hippocampus; *R*=0.90, entorhinal cortex). To further investigate whether Notch1 and APP, which are both targets of gamma secretase, are present in fibrillary aggregates, we performed double immunolabeling of Notch1 with APP and counterstained for Thioflavin T. We determined that Notch1 and APP are colocalized in some but not all Thioflavin T positive structures (Fig. 4d’, white arrows). The partial colocalization of Notch1 with APP was previously observed in the brains of 22 months old PolyI:C mice (Additional file 2: Figure S2C).

### Notch1 localizes in NFTs

**Fig. 5:**
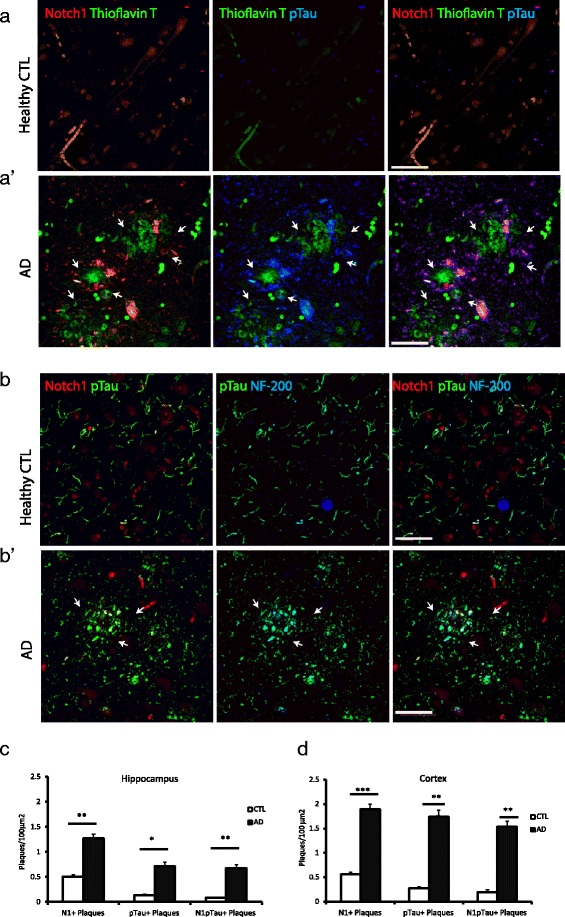
Notch1 colocalizes with p-Tau in fibrillary plaques. **a**–**a**’) In the entorhinal cortex of demented patients, Notch1 costaining with p-Tau surrounds Thioflavin T labeled plaques (arrows in **a**’), whereas in the healthy control (**a**) there is no presence of aberrant aggregates. **b**–**b**’) In the AD cortical tissue, Notch1 is co-expressed with p-Tau in NF-200 positive neurofibrillary filaments (arrows in **b**’), whereas there is no apparent colocalization in the healthy control tissue (**b**). **c**–**d**) Bar graphs indicating the amount of plaques either in the hippocampus (**c**) or in the entorhinal cortex (**d**) positive for Notch1, p-Tau and double stained. *= *p*<0.05, **= *p*<0.01, ***= *p*<0.001. Error bars are SEM. In all panels scale bars are 40 *μ*m

**Fig. 6:**
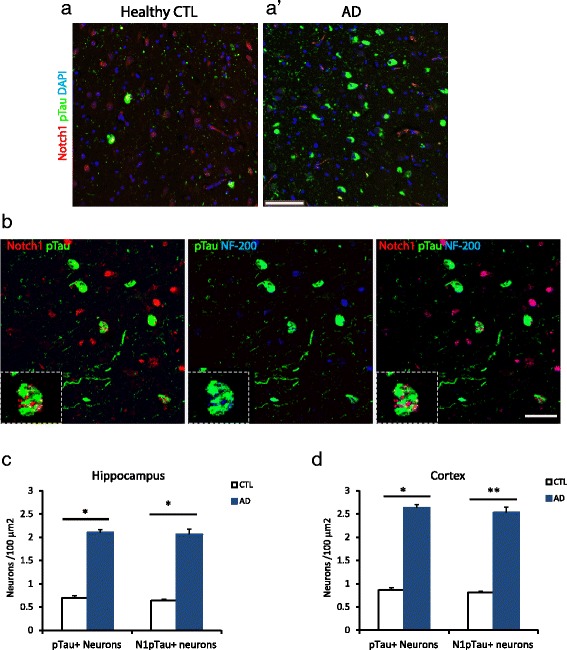
Intracellular depositions of Notch1 and p-Tau are increased in AD degenerating neurons. **a**–**a’**) Double immunostaining showing the enhanced number of damaged neurons labeled with p-Tau and partially with Notch1 in AD and healthy control cortical tissue. **b**) Triple staining showing the co-expression of Notch1 and p-Tau in neurofilament positive degenerating cortical neurons (insert). **c**–**d**) Bar graphs indicating the counting of degenerating neurons positive for p-Tau or double positive for p-Tau and Notch1, either in the hippocampus (**c**) or in the entorhinal cortex (**d**). *= *p*<0.05, **= *p*<0.01, Error bars are SEM. Scale bar in (**a’**) is 100 *μ*m and in (**b**) 40 *μ*m

Based on the pattern of Notch1 in AD brains, we hypothesized that this protein is present in fibrillary tangles. Therefore, we performed double immunolabeling with Notch1 and phosphorylated Tau (p-Tau) and counterstaining with Thioflavin T. We observed a nearly complete overlap of Notch1 with p-Tau in plaque-like structures (Fig. 5). As previously observed (Fig. 4a’), the core of the plaques is stained for Thioflavin T only with its radius positive for all the three markers (Fig. 5a’, arrows). As expected p-Tau and Thioflavin T show low expression in the age-matched controls (Fig. 5a). To further validate that Notch1 is localized in dystrophic neurites, we performed co-immunolabeling with p-Tau and the heavy neurofilament, NF-200, which are the characteristic components of neurofibrillary tangles (NFTs) [39] (Fig. 5b-b’). We observed a great overlap among the three proteins in plaques and tangles distributed in the parenchyma of the entorhinal cortex (Fig. 5b’) and dorsal hippocampus (data not shown). In healthy controls, the three proteins are present for the majority in filaments and not aggregates, with rare occurrence in plaques and tangles (Fig. 5b–d). The presence of Notch1 in filamentous structures was marked also in 22 months old PolyI:C mice (Additional file 2: Figure S2C, white arrows). Quantification of Notch1 and p-Tau positive plaques indicates that all plaques presenting p-Tau have positivity for Notch1 in the dorsal hippocampus, but not viceversa (Fig. 5c). On the other hand, in the entorhinal cortex the majority of p-Tau plaques have also Notch1 (Fig. 5d). Overall, correlation analysis confirms the coincidence of Notch1 and p-Tau in plaque depositions in both areas (*R*=0.76, dorsal hippocampus; *R*=0.82, entorhinal cortex). Furthermore, in all analysis performed in the regions of interest, we observe that Notch1 positive plaques tend to be supranumerous to the plaques positive for p-Tau (Fig. 5c and d), Thioflavin T (Fig. 4b and c) or A *β*42 (Fig. 1f and g). This can be explained by the fact that Notch1 can be found in a variety of depositions as well as cellular species. Yet, it remains possible that Notch1 aggregates hallmark a novel type of plaques or label protein deposits following neuronal demise. Besides the appearance of Notch1 in aggregates disseminated in the brain parenchyma, we could observe that, in AD, the majority of the degenerating neurons expressing high levels of p-Tau were also positive for Notch1 (Fig. 6a’). This overlap was also present in scattered neurons from the healthy controls (Fig. 6a). Evidence of ongoing neurodegeneration in the double positive Notch1 and p-Tau neurons is indicated by the displacement of NF-200 to the cell soma (Fig. 6b). Similarly to the appearance of Notch1 in NFTs, the three proteins colocalize indicating that Notch1 is also accumulating in the dying neurons (Fig. 6b, insert). Analysis of the degenerated neurons, as indicated by the presence of p-Tau, show also an accumulation of Notch1 in both dorsal hippocampus (Fig. 6c) and cortex (Fig. 6d). Furthermore, in the entorhinal cortex, we observed the presence of Notch1 protein in neurons with polynucleate morphology in both AD (1.6 % ± 0.6) and control samples (0.7 % ± 0.3) (*p*=0.2) (Additional file 5: Figure S5A and B). The number of neurons with polynucleate morphology tended to be higher in the AD patients, but did not reach significance (F1,8= 1.55, *p*=0.2). On the other hand, the percentage of polynucleate neurons in the CA pyramidal layer was indistinguishable between AD and controls. The localization of Notch1 in those cells suggests, as previously described, that Notch1 may be involved in cell cycle re-entry [30], a mechanism underlying progressive neurodegeneration.

### Notch1 levels, but not activation, are increased in AD

**Fig. 7:**
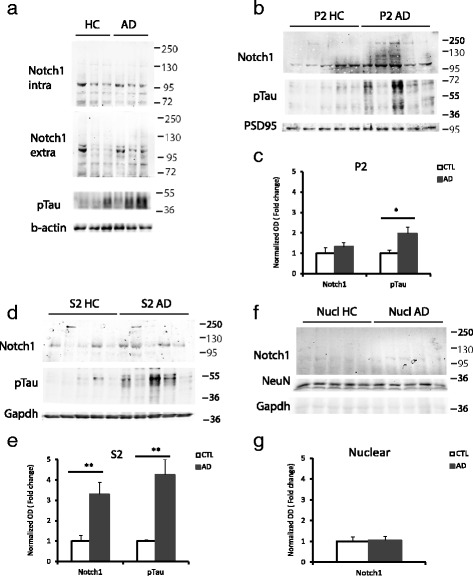
Notch1 levels in the entorhinal cortex of AD patients. **a**) Representative Western Blot analysis on whole cell lysate indicates no difference in Notch1 levels, represented by the extracellular and the intracellular component, between healthy controls and AD patients. In contrast, p-Tau is increased in AD lysates. **b**–**e**) Western blot on synaptosomal fractions shows the unchanged expression of Notch1 in the synaptic (P2) component (*p*=0.2) of AD samples as compared to the controls (**b** and **c**). On the other hand, in the cytoplasmic compartment (S2) Notch1 levels are increased (*p*=0.007) in AD samples as compared to the controls (**d** and **e**). In addition, in both fractions, the levels of p-Tau are augmented in AD samples, (P2, *p*=0.02; S2, *p*=0.002) as compared to the age matched controls (**c** and **e**). **f**–**g**) Immunoblotting (**f**) and bar graph (**g**) on nuclear fraction indicates no difference in Notch1 expression between AD and healthy controls (*p*=0.25). *= *p*<0.05, **= *p*<0.01. Error bars are SEM

**Fig. 8:**
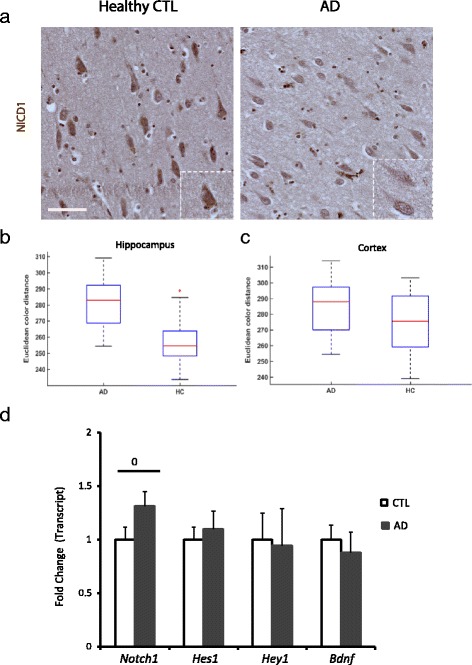
Notch1 signaling in AD patients. **a**) Immunohistochemistry using an antibody specific for NICD1 shows that in AD cortices the activation of Notch1 in pyramidal neurons is lower as compared to the healthy controls (inserts). **b**–**c**) Box plots summarizing the NICD signal shows a significant difference in immunoreactivity in (**b**) hippocampal and (**c**) cortical neurons between AD and healthy CTLs. **d**) Bar graph showing the fold change in transcript levels of Notch1 and some target genes, such as *Hes1*, *Hey1* and *BDNF*. Only the mRNA of Notch1 is near to significance (0 = *p*=0.06). Error bars are SEM. Scale bar in (**a**) is 50 *μ*m

In order to assess the expression of Notch1 in the AD brain tissue, we conducted Western Blot analysis on whole cell lysates as well as crude synaptosomal, cytosolic and nuclear fractions from entorhinal cortices. In the whole cell lysate preparation, we observed an increase in p-Tau in the AD samples (Normalized OD: 4.5 ± 1.8) as compared to the age-matched controls (OD: 1 ± 0.2) (*p*=0.04) (Fig. 7a). However, Notch1 levels appeared unchanged, as detected by two antibodies: one recognizing the C-terminal tail (Normalized OD: 1 ± 0.7 versus 0.8 ± 0.2; *p*=0.9) and another one against the extracellular domain (Normalized OD: 1 ±0.3 versus 1.5 0.7; *p* = 0.49) (Fig. 7a). Being Notch1 expressed by a variety of cells populations in the brain and in different cellular compartments, it remained possible that alteration in Notch1 localization could be observed using cellular fractionations. In the synaptosomal membrane fractions, Notch1 levels as measured by the 100KDa band appear unchanged, whereas p-Tau was increased (Fig. 7b and c). In contrast, in the cytosolic compartment we observe an increase in both Notch1 and p-Tau in ADs as compared to healthy controls (Fig. 7d and e), suggesting that the turnover of both proteins is affected in AD. Interestingly, in the nuclear fraction, the 100KDa band corresponding to the Notch1 intracellular domain (NICD) is represented at very low levels in both healthy controls and AD samples. (Fig. 7f and g). To further explore the extend of Notch1 processing in neurons, we performed IHC using an antibody specific for the cleaved Notch1 and quantified the signal in the soma of cortical (Fig. 8a) and hippocampal neurons (data not shown). We observed that NICD was considerably lower, showing higher euclidean color distance, in the neurons from the AD patients as compared to the healthy controls (*p*<0.001 for both regions) (Fig. 8b and c). Furthermore, the levels of canonical target genes, *Hes1* and *Hey1*, and indirect targets *BDNF* from RNA obtained from whole tissue preparation were indistinguishable between ADs and CTLs (Fig. 8d). This suggests that overall Notch1 signaling is not increased in the brains of AD patients. On the other side, from our whole cell preparation, we observe a 30 % increase in Notch1 transcripts, which is near to significance (*p*=0.06) (Fig. 8d). This may reflect an imbalance in Notch1 in more than one cell type in the AD brains.

### Notch1 levels are reduced in the CSF of AD patients

**Fig. 9:**
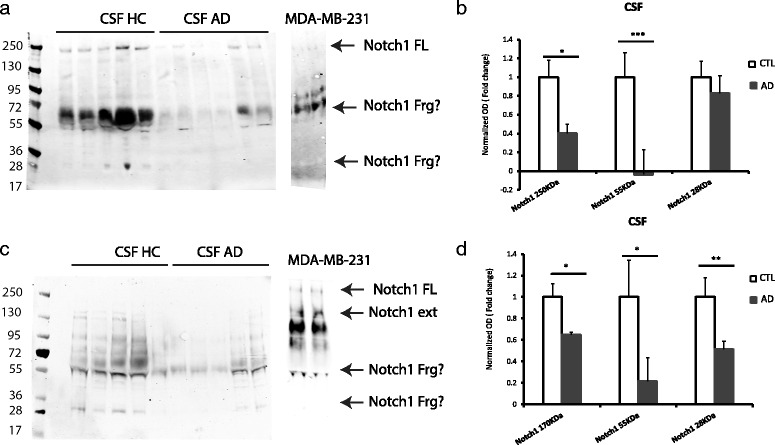
Notch1 fragments are less represented in the CSF from AD patients. **a**) Immunoblotting on CSF reveals that the full length (FL) of Notch1 is decreased in AD samples as compared to healthy controls. Moreover, other fragments of the protein around 55 and 28 KDa appear decreased in AD samples. **b**) Bar graph showing the pattern of expression of Notch1 FL and the smaller fragments in AD samples as compared to healthy controls. **c**) Immunoblotting on CSF showing the diminished level of the extracellular portion of Notch1 in AD samples as compared to age matched controls. Also with this antibody we detected fragments, either around 55 or 28 KDa, which are reduced in AD CSF. **d**) Bar graph showing the reduction of Notch1 extracellular domain and the smaller truncations of the receptor in AD samples. *= *p*<0.05, **= *p*<0.01, ***= *p*<0.001. Error bars are SEM

It is established that a variety of proteins are cleared from the brain into the cerebrospinal fluid [40]. When the proteins start accumulating into the brain parenchyma, filtration through the CSF is reduced. As a result in AD patients many proteins, with the exception of Tau, which is overexpressed in tangles, are reduced. In our study, we investigated whether Notch1, which aggregates in plaques and NFTs, is subject to differential clearance in AD patients versus age-matched controls. Using two different antibodies recognizing the extracellular and the intracellular domain of Notch1, as well as the supernatant from the human breast cancer cell lines (MDA-MB-231) as control, we observed that Notch1 is present in CSF and cellular supernatant in various forms. Specifically, we detected: i) the full length protein around 250KDa, ii) truncation forms between 72KDa and 55KDa in both CSF and cellular supernatant, whereas the iii) small fragments around 28KDa were only present in the CSF (Fig. 9a). This suggests that Notch1 may be released or leaked out as proteolytic fragments. All these protein forms are reduced in AD samples as compared to healthy controls (Fig. 9b). Presence of the full length protein or the extracellular domain is confirmed by the blot using the antibody specific for the extracellular domain (Fig. 9c) and could be identified as a band between 130 and 250KDa, as well as additional bands around 55 and 33KDa. The signal ratio between the controls and AD indicates that Notch1 extracellular portions are reduced in AD as compared to controls (Fig. 9d). These data suggest that in the CSF several fragments of Notch1 are present and that there is depletion of those protein species in the CSF of AD patients as compared to healthy controls.

### Notch1 aberrant expression in AD livers

**Fig. 10:**
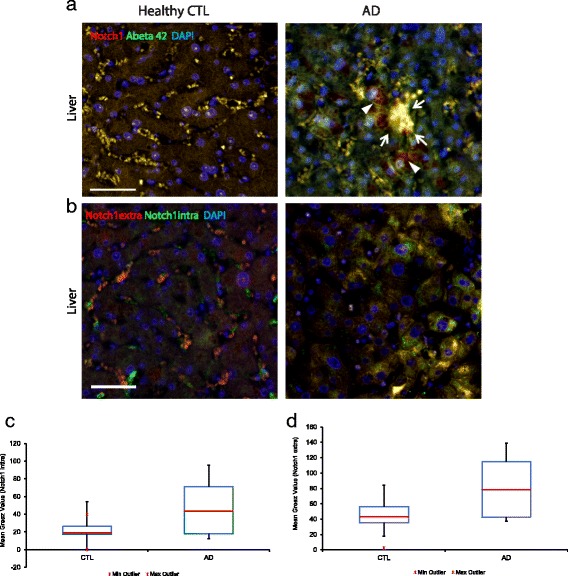
Altered Notch1 expression and deposition in the liver of AD patients. **a**-**b**) Immunofluorescence on liver sections. **a**) Notch1 and A *β*42 positive aggregates (arrows) in the hepatic parenchyma of AD patients, but not in controls. Moreover, Notch1 is highly expressed in the cytosol of several hepatocytes (arrowsheads). **b**) Antibodies specific for the extracellular and intracellular portions of Notch1 strongly label hepatocytes in the AD liver as compared to the healthy control section. **c**) Box plot summarizing the mean grey value intensity of the Notch1 intracellular signal in AD and CTL hepatocytes. **d**) Box plot summarizing the mean grey value intensity of the Notch1 extracellular signal in AD and CTL hepatocytes. The scale bars are in (**a**) and (**b**) 50 *μ*m

To further investigate whether the accumulation in the brain correlates with peripheral alteration in Notch1 turnover, we performed immunohistochemical analysis on liver sections from AD and healthy patients comparing Notch metabolism with amyloid processing. The presence of Notch1 and A *β*42 depositions was visible in the liver parenchyma of 3 out of 5 AD patients (Fig. 10a, white arrows). Hepatocytes surrounding the extracellular plaques showed overexpression of Notch1 (Fig. 10a, white arrowheads). Plaques were seen in sections revealing a significant increase (more than 2 folds) in Notch1 expression and activation, as indicated by the Notch1 extracellular and Notch1 intracellular labelings, respectively (Fig. 10b) (*p*<0.05 for both labelings). Box plot analysis indicates the distribution of Notch1 extracellular (Fig. 10d) and Notch1 intracellular (Fig. 10c) domains in AD and healthy controls. These results suggest that, similarly to A *β*42, alteration of Notch1 in the liver may contribute to plaque formation peripherally. These aberrant peripheral aggregates could represent a natural sink for misfolded proteins [41] including Notch1.

## Discussion

In this work, we show for the first time that Notch1 accumulates in fibrillary plaques and is remarkably associated to NFTs in the brains of sporadic AD patients. Similarly, in the neocortex of PolyI:C mice, which recapitulate aspects of sporadic AD [11], we observe a progressive accumulation of Notch1 in the brain parenchyma. In both demented patients and PolyI:C mice, Notch1 depositions show a neuroinflammatory response characterized by the presence of reactive microglia. Such delocalization of Notch1 to plaques and tangles is concomitant with a reduction in Notch1 expression and activation in cortical and hippocampal neurons. Moreover, a consistent reduction in Notch1 protein levels is detected in the CSF from AD patients as compared to healthy controls. This suggests a retention of the protein in the brain milieu. Last, we provide evidence of an accumulation of Notch1 in amyloid plaques’ formations in the liver, which may represent a repository contributing to the progressive neurodegeneration. Despite the limited number of specimen used in this study, the results are strikingly consistent within each group and indicate that Notch1 aggregations may be considered an additional hallmark of AD.

### Notch1 association to NFTs

Cytoskeletal integrity and plasticity is determined by the constant turnover of proteins associated to microtubules such as Tau. In AD, the cytoskeletal structure is strongly destabilized by the overwhelming abundance of phosphorylated Tau, which collapses axonal integrity and contributes to the relocalization of Tau from the axonal to the somatodendritic compartment. Moreover, transynaptic diffusion of Tau protein is thought to underlie the progressive spread of NFTs in the brain [42]. In this study, we observed that Notch1-positive aggregates highly correlate with NFTs as well as amyloid neuritic plaques. The colocalization of Notch1 and p-Tau is also found in healthy controls, but at considerable lower rate as compared to AD patients. This suggests a functional interaction between the Notch1 membrane receptor and microtubules-associated proteins, as previously proposed [43, 44]. Studies on primary cortical neurons have shown that Notch regulates neurites’ extension and morphology [45, 46]. In addition, it was shown that Notch activation favors microtubules stabilization, reduction of neurite arborization and varicosities, whereas Notch1 inhibition can revert these events promoting cytoskeletal plasticity [44]. Interestingly, one of the critical protein in balancing Tau turnover is Pin1 [47, 48], a direct target of Notch1 in breast cancer cells [49, 50]. Our own unpublished studies confirm the dependence of Pin1 on Notch1 activity and show that hippocampal neurons devoid of Notch1 display reduced Pin1 levels along with an increase in Tau phosphorylation (data not shown). Notably, the accumulation of Notch1 in NFTs, in AD patients, does not correlate with increased signaling but rather the opposite, as indicated by reduced NICD1 in pyramidal neurons. Thus, it is possible that a dysfunction in Notch1 signaling may contribute to microtubule instability through Pin1. It remains to be understood how and why the full membrane receptor accumulates in NFTs and whether this interaction contributes to cytoskeletal disassembly. Overall, our observations suggest that Notch1 delocalization to NFTs may have significant implication for neurodegeneration.

### Notch1 signaling is reduced in AD neurons

One of the late symptoms of AD is represented by the memory impairment, the progressive disability in speaking and altered behavior. It is established that hippocampal plasticity, which is essential for memory and emotions, is strongly affected in AD. One of the main transduction pathways contributing to memory establishment is Notch1. This signaling receptor is highly expressed in principal neurons belonging to neuronal networks involved in information processing [25] and memory formation [22, 24]. Loss of Notch1 from flies to rodents’ brains affects memory and learning [51]. Furthermore, recent work from our own group has shown that Notch1 interacts with ApoER2 and NMDAR, which are critical mediators of synaptic plasticity with an established role in AD progression. Through this interaction, CREB-dependent signaling is facilitated and contributes to the molecular basis of memory formation [52]. These studies, hint at a possible involvement of Notch1 signaling in the memory demise characterizing AD. To corroborate this hypothesis, our own study shows that Notch1 levels and activity are significantly reduced in principal neurons of the hippocampus and cortex. The absence of a reduction in canonical targets, *Hes1* and *Hey1*, could be explained by the analysis conducted on whole cell tissue preparations including both neurons and glia. Overall, downregulation of Notch1 may impact synaptic plasticity and affect downstream effectors of memory [51]. These results support the notion that dysfunction in Notch1 expression/activity in AD may contribute to the displacement/accumulation in plaques or NFTs and reduced signaling in neurons.

### Notch1 aggregates may contribute to neuroinflammation

Several studies suggest that one of the main contributing factors to the onset and the progression of AD is neuroinflammation. The first stages of sporadic AD are triggered by an excessive or chronic inflammatory stimulus, which induces an increase in misfolded proteins, a reduction in their clearance and a destabilization of microtubules. This event impairs the physiological cellular trafficking along the axonal processes, causing an aberrant accumulation of proteins and other molecules in the axons length, forming swollen areas known as “axonal varicosities” and a leakage of these proteins from the axonal compartment to the brain parenchyma [53]. Leaked proteins further cause neuroinflammatory responses triggering progressive astrogliosis and microgliosis in the interested area [53]. In our study, we observe that, in AD patients, Notch1 depositions in the brain parenchyma are surrounded by glial cells suggesting that Notch1 may be released from neurons and contribute to the neuroinflammatory events aimed at protein clearance [54]. Moreover, Notch1 can be found in both glial types suggesting an involvement of this pathway in the modulation of the inflammatory activity. It is reported that all the Notch components are expressed and active in microglia and the Notch pathway modulates the inflammatory potential of these cells [31, 38]. Interestingly, in the PolyI:C model of neuroinflammation, activated microglia are also positive for Notch1 hinting at an ongoing inflammatory process. On the other hand, in AD brains, astroglia is characterized by high level of nuclear Notch1, which is a well established proliferative factor for astrocytes [55]. This implicates Notch1 signaling in progressive astrogliosis occurring in AD. Thus, in the CNS, Notch signaling components are expressed in different cellular populations: i) neurons, which express both ligand and receptor proteins [21, 22], ii) astroglia, which mainly present the ligand Jagged1 [21] and iii) microglia, which abundantly express Notch1 [56]. This evidence suggests a complex interplay among different cell types within the brain. Such mechanisms, which need to be yet uncovered, underlie the importance of Notch activity in modulating brain homeostasis. Hence, an impairment in this cascade may disrupt the cellular crosstalks and contribute to an uncontrolled inflammatory response observed in AD.

### Evidence of Notch1 alteration in the CSF of AD patients

One of the diagnostic methods to detect and monitor the progression of AD is the sampling of CSF. Despite the variety of proteins, which are present in the CSF [40], the best characterized markers are A *β*42 and Tau [57]. The measurement of the ratio between the two proteins establishes the advancement of the disease. Typically, when A *β*42 is accumulated in plaques, as AD progresses, levels in the CSF decline. On the other hand, neuronal demise contributes to the release of Tau in the CSF. Based on the evidence that Notch1 accumulates in plaques, we addressed whether levels in the CSF were changed in AD as compared to healthy controls. We observed that Notch1 is present in the liquor as full protein, extracellular fragment and smaller truncations around 55KDa and 28KDa. Former studies have detected the presence of transmembrane protein species, like APP and Presenilin1, in the CSF [37, 58]. Interestingly, extrusion of Notch1 as full receptor and extracellular portion could be also observed in the supernatant of human breast cancer cells. This indicates that either exocytosis of transmebrane proteins through exosomes is constitutive of mammalian cells or that such proteins are freed from dying cells. Moreover, smaller truncations of Notch1 were observed in the CSF samples only. Interestingly, a circulating proteosome has been recently detected both in plasma and CSF [59, 60]. This raises the possibility that Notch1 may undergo proteolysis at specific sites. Alternatively, those fragments could represent insoluble aggregates of misfolded Notch1 peptides. It has been previously shown that FAD mutations of Presenilins can generate longer N *β* peptides with more lipophylic properties [61]. It remains possible that, in sporadic AD, a dysfunction in gamma secretase activity, can lead to an increase of N *β* lipophylic species, which have the potential to form insoluble aggregates. Nevertheless, independently of the size of the fragments, all Notch1 forms were consistently less represented in the CSF of AD patients. This supports the notion that Notch1 is accumulating in the brain and may contribute to aberrant protein aggregates.

### Aberrant Notch1 in the liver of AD patients

Clearance of misfolded protein occurs both centrally and peripherally. The liver represents the principle site of peripheral clearance and it is reported that in AD such mechanism is affected [62]. A *β*42 fragments and possibly other lipophylic peptides can cross the blood-brain barrier (BBB) by binding to the lipoprotein receptor-related protein 1 (LRP1) and the very-low density lipoprotein receptor (VLDLR), which export these peptides from the brain to the blood and finally to the liver. In opposite direction, the receptor for advanced glycation end products (RAGE) determines the passage of A *β*42 from the blood to the brain [62]. In AD, the levels of the proteins involved in the efflux of A *β*42, from the brain to the blood, are decreased, while the expression of RAGE is increased, reinforcing the hypothesis on the primary role that peripheral clearance has in the removal of A *β* species [63]. Moreover, in the liver, the hepatic isoform of LRP contributes to the elimination of these aberrant peptides, involving several proteases including the insulin degrading enzyme (IDE) [64]. Interestingly, IDE has been identified as an indirect target of Notch1 [65]. All Notch receptors and ligands are expressed in the liver and Notch signaling plays a fundamental role in the metabolic homeostasis and is aberrantly induced in obesity-related liver diseases [66]. Based on these evidences, we investigated whether Notch1 was differentially expressed in the liver of sporadic AD patients. We show that Notch1 localizes in amyloid deposits in the hepatic parenchyma and that such aggregates are surrounded by hepatocytes with increased Notch1 expression. In the same patients, a general increase in Notch1 expression/activity was detected in the liver. Previous studies indicate that insulin resistance, as in diabetes, can increase Notch1 signaling [66]. Moreover, a mechanistic interaction between Notch and insulin signaling has been previously reported [67–69]. Notably, there is a strong correlation between diabetes and AD [70] and insulin-resistance is though to be one of the major risks for developing dementia [71]. Thus, it is possible that progressive alteration in insulin turnover in the liver causes Notch1 over-activation with aberrant release of Notch1 fragments. These species may lead to peripheral plaque formation and contribute to a sink in misfolded protein, including Notch1, which could be recycled to the brain.

## Conclusions

This study sparks unprecedented evidence of an involvement of Notch1 in sporadic AD. In the human brain tissues examined, entorhinal cortex and dorsal hippocampus, we observed a profound delocalization of Notch1 from neurons to extracellular aggregates and NFTs. It remains to be established whether this phenomenon extends to other brain regions and whether it follows the spread of fibrillary tangles according to the Braak staging. Yet, the present data suggest that Notch1 may be involved, as previously described from flies to mammals, in cytoskeletal integrity and plasticity and participates, if altered, to neurodegeneration. In contrast, the levels of Notch1 in pyramidal neurons of the hippocampus and cortex were significantly reduced implicating that loss of Notch1 in neurons may have effect on synaptic plasticity and memory processes, which are significantly impaired in AD. Moreover, we demonstrate that Notch1 is cleared in the CSF and that such clearance, similarly to A *β*, is drastically reduced in AD patients suggesting an accumulation of Notch1 in the brain parenchyma. Finally, we provide preliminary evidence for a peripheral accumulation of Notch1 in the liver in amyloid aggregates accompanied by an aberrant increase of Notch1 in hepatocytes. All together these data suggest that Notch signaling may be considered among the critical pathways involved in the pathobiology of sporadic AD. A further comparative analysis of Notch1 expression/processing in different neurodegenerative diseases in humans is necessary to establish whether Notch1 may be considered a specific therapeutical biomarker for AD.

## Additional files

Additional file 1
**Figure S1.** Schematics for immunohistochemistry analysis. **A**-**C’**) Workflow of the analysis performed to quantify the chromogen signal of Notch1 and NICD1 in neurons. **A**) Representative image of a brain tissue slice stained for Notch1 and counterstained with Nissl displays, for clarity, marked boundaries of the ROIs analyzed in the study [red= dorsal hippocampus (CA1-CA2 regions) and green = entorhinal cortex]. **B**) Example of randomly selected area from the entorhinal cortex, which was imported in TrakEM2. Neuronal somas selected for analysis are highlighted in yellow. **B’**) Representative mask of the selected cells obtained with TrakEM2 and imported into Matlab. **C**) Processed signal of Notch1 in a pyramidal neuron is colorized in green. **C’**) Corresponding histogram representing RGB color thresholds as assigned by the program. **D**-**E"**) Workflow of the analysis performed on immunofluorescent stained sections to quantify the number of plaques and neurons positive for Notch1 and other markers. **D**) Representative image of a brain section fluorescently labeled for Notch1, p-Tau and counterstained with DAPI. Marked boundaries indicate the ROIs analyzed [red= dorsal hippocampus (CA1-CA2 regions) and green = entorhinal cortex]. **E**) Example of a randomly selected area from the cortex divided in 24 squares (100 *μ* *m* ^2^/each), in which the countings were carried out for the analysis. Area splitted according the fluorophore corresponding to **E’**) Notch1 and **E"**) p-Tau. Scale bars in **A** and **D** are 5 mm and in **E**-**E"** 100 *μ*m. (PDF 1,326 kb)

Additional file 2
**Figure S2.** Ectopic Notch1 expression in the brain of 22 months old PolyI:C mice. **A**-**A"**) Brain sections from aged mice, injected prenatally either with NaCl or PolyI:C are immunolabeled with Notch1. **A**) Notch1 expression is increased in the brains of the PolyI:C mice. **A’**) Zoom in of the hippocampal formation shows visible clumps positive for Notch1. **A"**) Larger magnification of the CA3 field shows a disrupted pattern of Notch1 expression in the neurons and Notch1 positive aggregates in the molecular layer in PolyI:C mice. **B**) Double immunofluorescence for Notch1 and the activated microglia marker, CD68, shows that, in PolyI:C mice, microglia are more abundant as compared to the age-matched controls and are intensively labeled for Notch1 (white arrowhead). **C**) Triple immunofluorescence staining for Notch1, APP and CD68 shows that Notch1 expression displays a different pattern in PolyI:C mice as compared to the controls. Moreover, Notch1-APP positive plaques (arrowheads) are surrounded by CD68-positive microglia (insert of a 4 fold magnification of the plaque on the right). Notch1 immunoreactive processes (arrows) are strongly labeled in the CA1 field of PolyI:C mice. Cx= cortex The scale bars are in **A** 1 mm, in **A’** 500 *μ*m, in **A"** and **C** 50 *μ*m and in **B** 25 *μ*m. (PDF 2825 kb)

Additional file 3
**Figure S3.** Distinctive patterns of Notch1 deposition in the brain parenchyma. Labeling of Notch1 (red) and A *β*42 (green) from an entorhinal cortex of an AD patient shows some Notch1 aggregates with no positivity for A *β*42 (white arrows). On the other hand, Notch1 (red) decorates the majority of A *β*42 plaques (light blue arrows). The scale bar is 40 *μ*m. (PDF 274 kb)

Additional file 4
**Figure S4.** Notch1 expression in fibrillary structures in the cortex. **A**) Variation of Fig. 3a": double immunolabeling for Notch1 intracellular and Notch1 extracellular shows that the two Notch1 domains overlap in fibrillary aggregates, whereas nuclei, as indicated by DAPI, are highly immunoreactive for the cytoplasmic form of Notch1. **B**) Labeling of Notch1 and Thioflavin T shows that, in AD patients, Thioflavin T positive fibrils are also marked for Notch1. Expression of Notch1 in the healthy controls is cytoplasmic and devoid of any Thioflavin T staining. The scale bars are in **A** 40 *μ*m and in **B** 100 *μ*m. (PDF 757 kb)

Additional file 5
**Figure S5.** Notch1 localization in neurons with polynucleate morphology. **A**) Chromogen immunolabeling shows that Notch1 intensively labels dividing neurons, as indicated by Nissl staining, highlighting the polynucleate morphology. **B**) Bar chart summarizing the countings of dividing cells in the hippocampus and cortex shows no significant difference between controls and demented patients. The scale bar in **A** is 5 *μ*m. (PDF 506 kb)
