# Erratum to: Notch1 hallmarks fibrillary depositions in sporadic Alzheimer’s disease

**DOI:** 10.1186/s40478-016-0360-1

**Published:** 2016-08-25

**Authors:** E. Brai, N. Alina Raio, L. Alberi

**Affiliations:** 1 Unit of Anatomy, Department of Medicine, University of Fribourg, Route de Gockel, 1, Fribourg, 1700 Switzerland; 2 Unit of Pathology, Department of Medicine, University of Fribourg, Route de Gockel, 1, Fribourg, 1700 Switzerland; 3 Swiss Integrative Center for Human Health, Passage du Cardinal, 13B, Fribourg, 1700 Switzerland

## Erratum

The authors of the original article [1] would like to make the readers aware that the acknowledgements section of this article was incomplete.

The updated text should read as follows:

We are particularly grateful to Prof. Francis (King’s College London, UK) for his valuable feedback on our submission for human samples to the Brain Bank for Dementia, UK. We would like to gratefully acknowledge all donors and their families for the tissue provided for this study. Human tissue samples were supplied by the Brains for Dementia Research programme, jointly funded by Alzheimer’s Research UK, the Alzheimer’s Society and the Medical Research Council, and sourced from the Oxford Brain Bank. The Oxford Brain Bank is also supported by the National Institute for Health Research (NIHR) Units.

We are sorry for any inconvenience caused.
