# Supplementary figures and images for: Notch1 hallmarks fibrillary depositions in sporadic Alzheimer’s disease
Source: Acta Neuropathol Commun. 2016 Jul 1;4:64. doi: 10.1186/s40478-016-0327-2 (PMC4929714; doi:10.1186/s40478-016-0327-2)

3VV[f[a` S^X\W#, 8Ygd\#

A

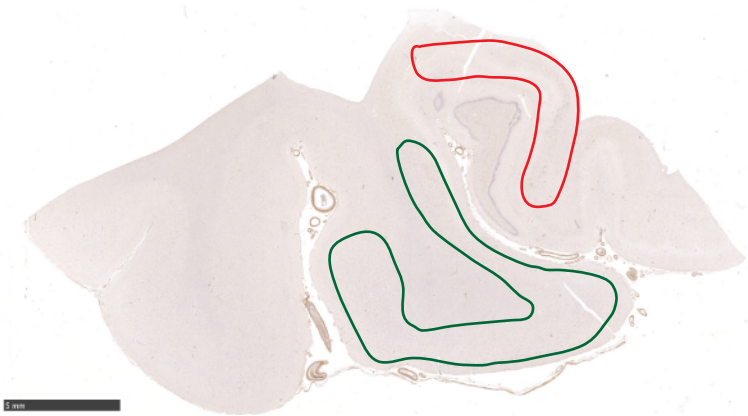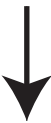

B

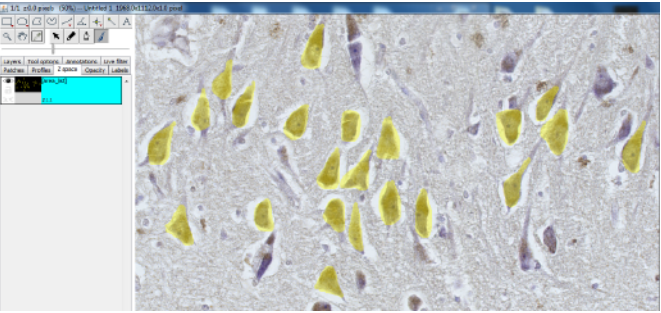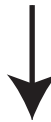

B'

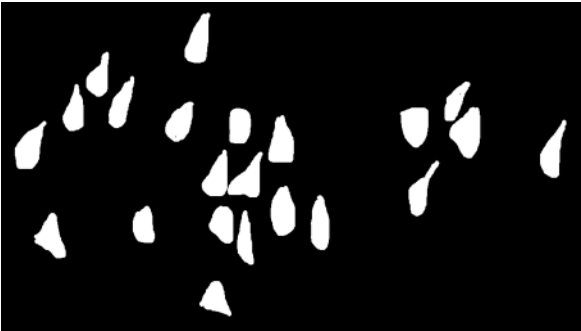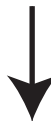

C

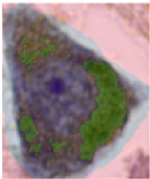

C'

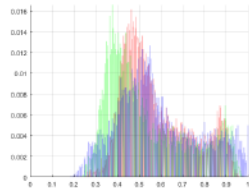

D

Notch1 pTau DAPI

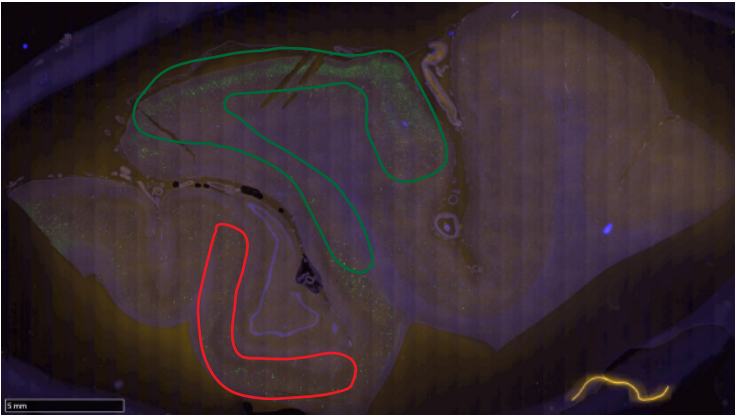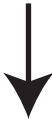

E

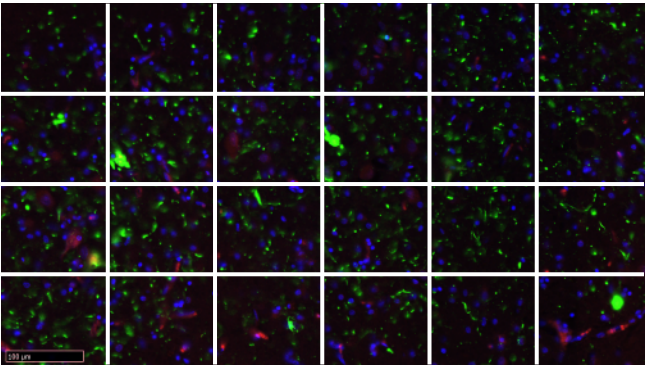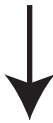

E'

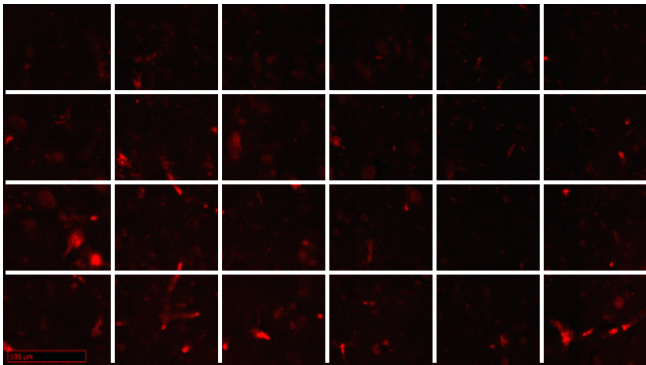

E''

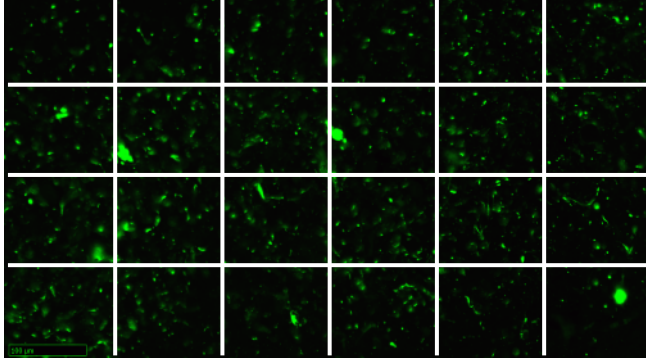

Supplement: Additional file 1 — Figure S1. Schematics for immunohistochemistry analysis. A-C’) Workflow of the analysis performed to quantify the chromogen signal of Notch1 and NICD1 in neurons. A) Representative image of a brain tissue slice stained for Notch1 and counterstained with Nissl displays, for clarity, marked boundaries of the ROIs analyzed in the study [red= dorsal hippocampus (CA1-CA2 regions) and green = entorhinal cortex]. B) Example of randomly selected area from the entorhinal cortex, which was imported in TrakEM2. Neuronal somas selected for analysis are highlighted in yellow. B’) Representative mask of the selected cells obtained with TrakEM2 and imported into Matlab. C) Processed signal of Notch1 in a pyramidal neuron is colorized in green. C’) Corresponding histogram representing RGB color thresholds as assigned by the program. D-E") Workflow of the analysis performed on immunofluorescent stained sections to quantify the number of plaques and neurons positive for Notch1 and other markers. D) Representative image of a brain section fluorescently labeled for Notch1, p-Tau and counterstained with DAPI. Marked boundaries indicate the ROIs analyzed [red= dorsal hippocampus (CA1-CA2 regions) and green = entorhinal cortex]. E) Example of a randomly selected area from the cortex divided in 24 squares (100 μ m 2/each), in which the countings were carried out for the analysis. Area splitted according the fluorophore corresponding to E’) Notch1 and E") p-Tau. Scale bars in A and D are 5 mm and in E-E" 100 μm. (PDF 1,326 kb) [file 40478_2016_327_MOESM1_ESM.pdf]

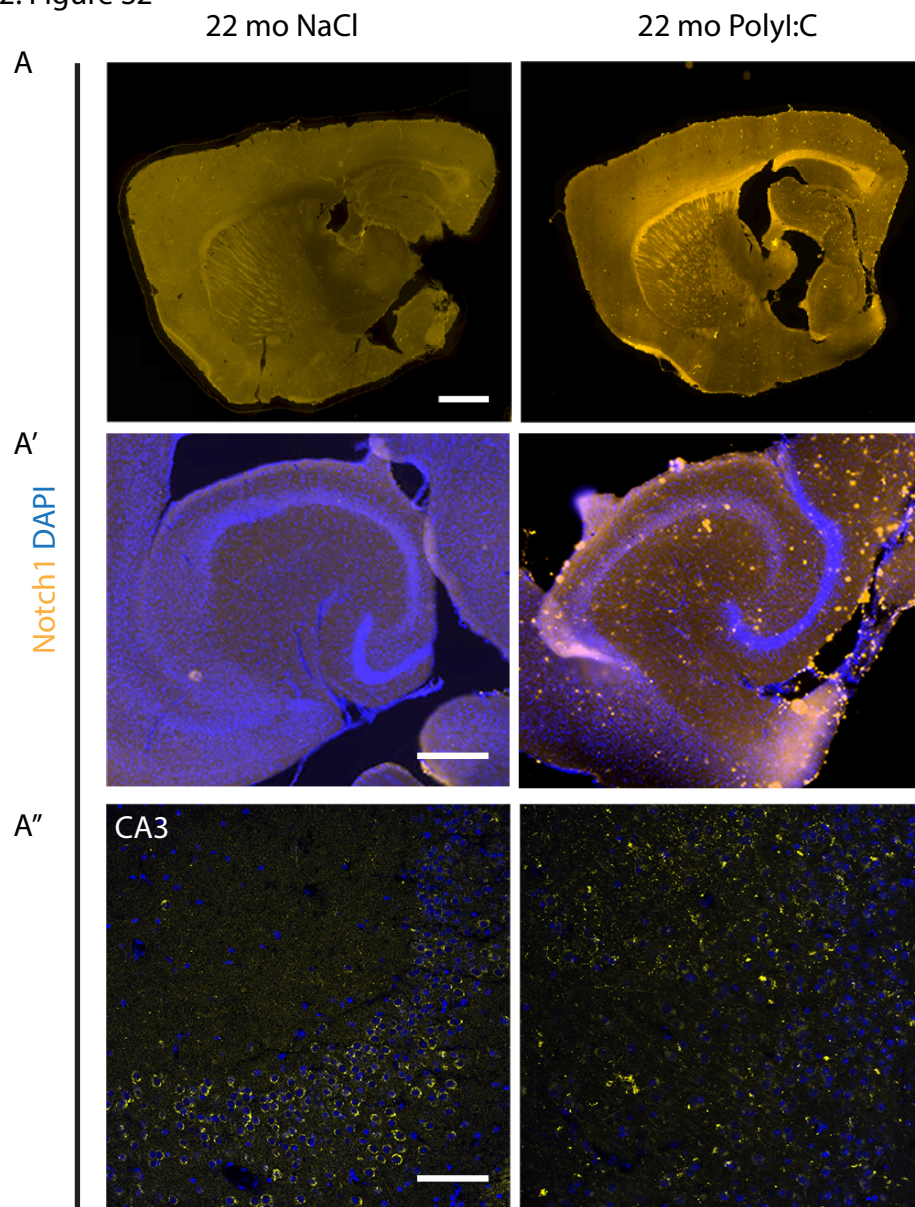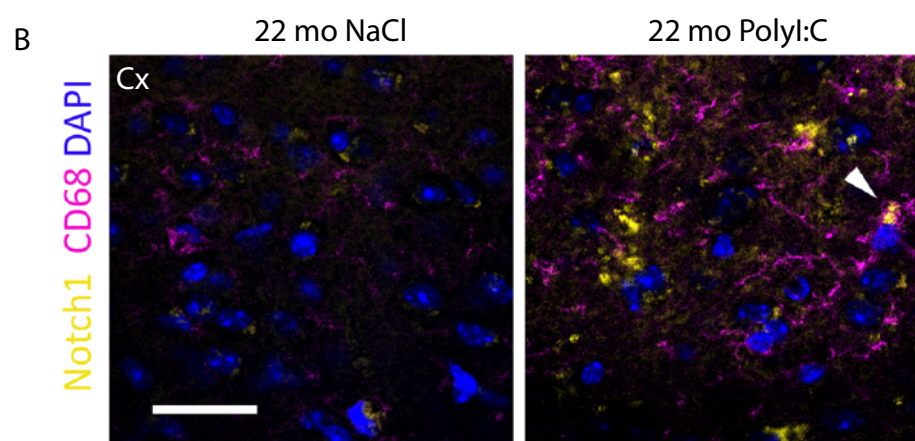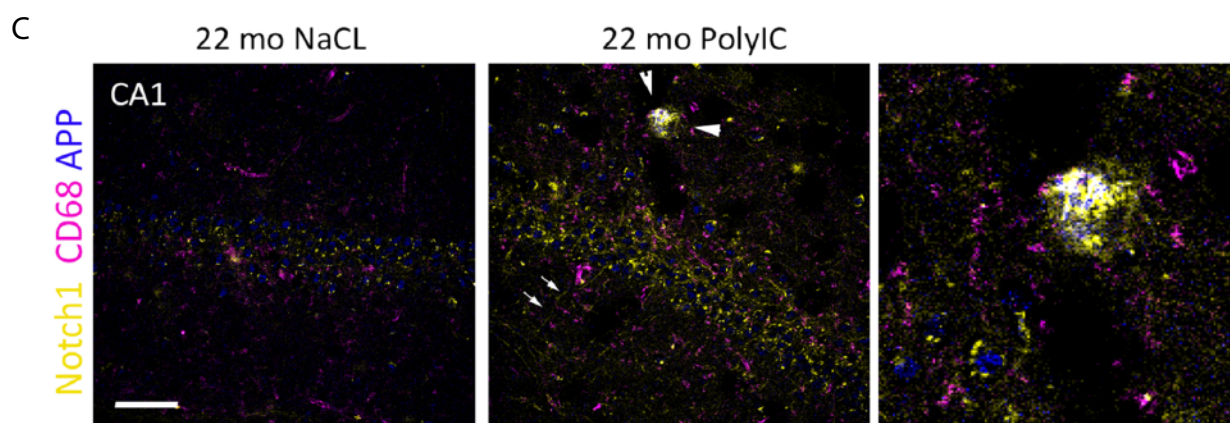

Supplement: Additional file 2 — Figure S2. Ectopic Notch1 expression in the brain of 22 months old PolyI:C mice. A-A") Brain sections from aged mice, injected prenatally either with NaCl or PolyI:C are immunolabeled with Notch1. A) Notch1 expression is increased in the brains of the PolyI:C mice. A’) Zoom in of the hippocampal formation shows visible clumps positive for Notch1. A") Larger magnification of the CA3 field shows a disrupted pattern of Notch1 expression in the neurons and Notch1 positive aggregates in the molecular layer in PolyI:C mice. B) Double immunofluorescence for Notch1 and the activated microglia marker, CD68, shows that, in PolyI:C mice, microglia are more abundant as compared to the age-matched controls and are intensively labeled for Notch1 (white arrowhead). C) Triple immunofluorescence staining for Notch1, APP and CD68 shows that Notch1 expression displays a different pattern in PolyI:C mice as compared to the controls. Moreover, Notch1-APP positive plaques (arrowheads) are surrounded by CD68-positive microglia (insert of a 4 fold magnification of the plaque on the right). Notch1 immunoreactive processes (arrows) are strongly labeled in the CA1 field of PolyI:C mice. Cx= cortex The scale bars are in A 1 mm, in A’ 500 μm, in A" and C 50 μm and in B 25 μm. (PDF 2825 kb) [file 40478_2016_327_MOESM2_ESM.pdf]

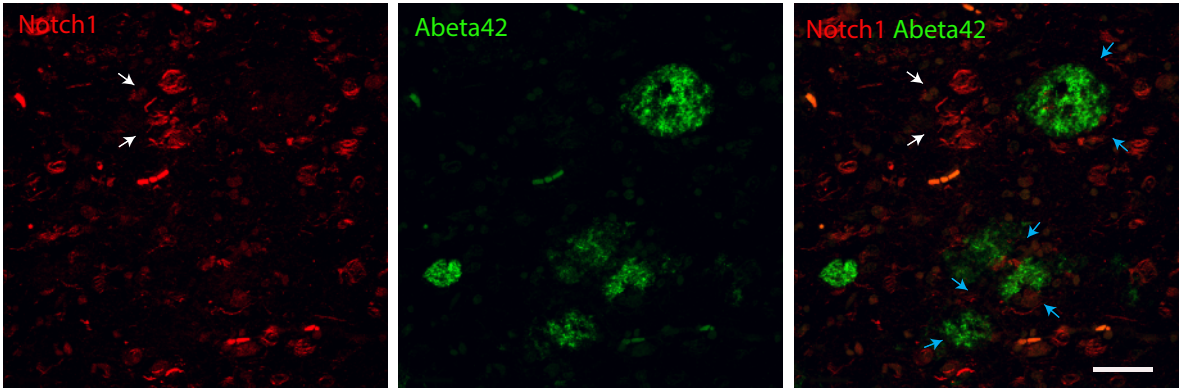

Supplement: Additional file 3 — Figure S3. Distinctive patterns of Notch1 deposition in the brain parenchyma. Labeling of Notch1 (red) and A β42 (green) from an entorhinal cortex of an AD patient shows some Notch1 aggregates with no positivity for A β42 (white arrows). On the other hand, Notch1 (red) decorates the majority of A β42 plaques (light blue arrows). The scale bar is 40 μm. (PDF 274 kb) [file 40478_2016_327_MOESM3_ESM.pdf]

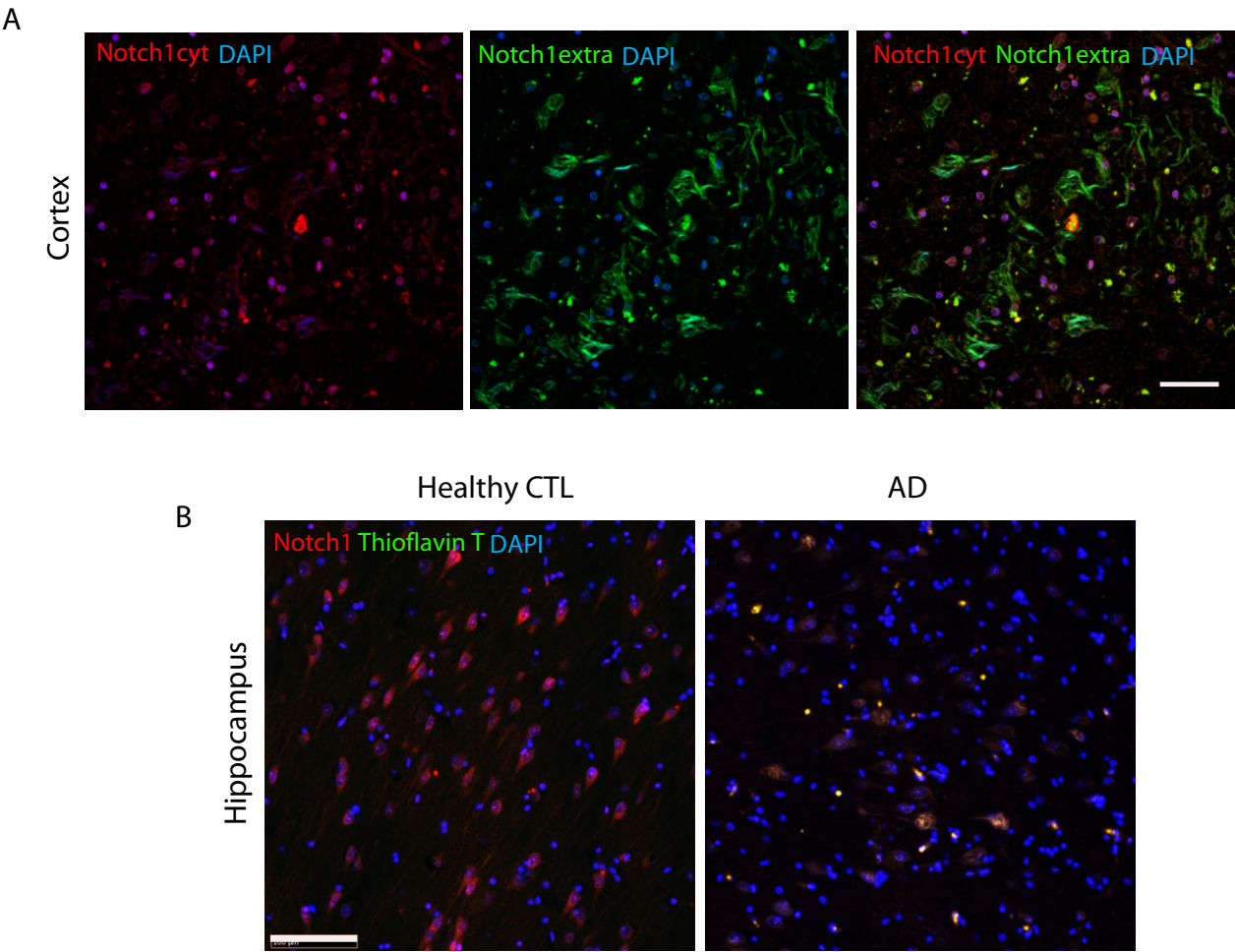

Supplement: Additional file 4 — Figure S4. Notch1 expression in fibrillary structures in the cortex. A) Variation of Fig. 3a": double immunolabeling for Notch1 intracellular and Notch1 extracellular shows that the two Notch1 domains overlap in fibrillary aggregates, whereas nuclei, as indicated by DAPI, are highly immunoreactive for the cytoplasmic form of Notch1. B) Labeling of Notch1 and Thioflavin T shows that, in AD patients, Thioflavin T positive fibrils are also marked for Notch1. Expression of Notch1 in the healthy controls is cytoplasmic and devoid of any Thioflavin T staining. The scale bars are in A 40 μm and in B 100 μm. (PDF 757 kb) [file 40478_2016_327_MOESM4_ESM.pdf]

A

Notch1

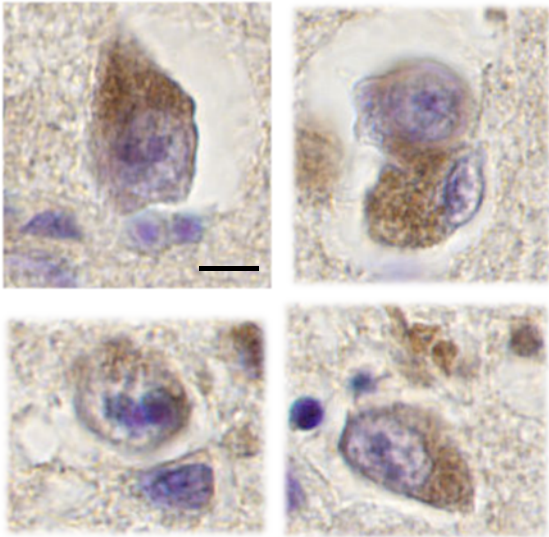

B

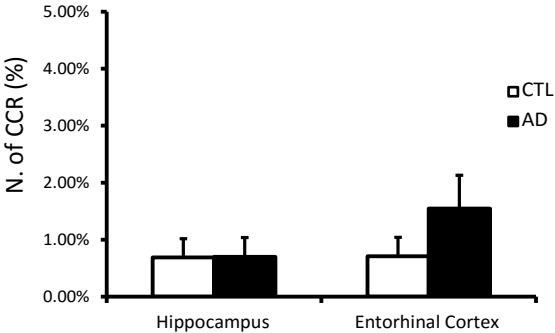

Supplement: Additional file 5 — Figure S5. Notch1 localization in neurons with polynucleate morphology. A) Chromogen immunolabeling shows that Notch1 intensively labels dividing neurons, as indicated by Nissl staining, highlighting the polynucleate morphology. B) Bar chart summarizing the countings of dividing cells in the hippocampus and cortex shows no significant difference between controls and demented patients. The scale bar in A is 5 μm. (PDF 506 kb) [file 40478_2016_327_MOESM5_ESM.pdf]
